# Exploring the Functional Brain Network of Alzheimer’s Disease: Based on the Computational Experiment

**DOI:** 10.1371/journal.pone.0073186

**Published:** 2013-09-03

**Authors:** YaPeng Li, Yuanyuan Qin, Xi Chen, Wei Li

**Affiliations:** 1 Image Processing and Intelligent Control Key Laboratory of Education Ministry of China, Wuhan, China; 2 Department of Control Science and Engineering, Huazhong University of Science and Technology, Wuhan, China; 3 Department of Radiology, Tongji Hospital, Tongji Medical College, Huazhong University of Science and Technology, Wuhan, China; “Mario Negri” Institute for Pharmacological Research, Italy

## Abstract

The purpose of this study is to explore the changes in functional brain networks of AD patients using complex network theory. In this study, resting-state fMRI datasets of 10 AD patients and 11 healthy controls were collected. Time series of 90 brain regions were extracted from the fMRI datasets after preprocessing. Pearson correlation method was used to calculate the correlation coefficient between any two time series. Then, a wide threshold range was selected to transform the adjacency matrix to a binary matrix under a different threshold. The topology parameters of each binary network were calculated, and all of them were then averaged within a group. During the evolution, node betweenness and the Euclidean distance between the nodes were set as control factors. Each binary network of healthy controls underwent evolution of 100 steps in accordance with the evolution rules. Then, the topology parameters of the evolution network were calculated. Finally, support vector machine (SVM) was used to classify the network topology parameters of the evolution network and to determine whether evolution results matched the datasets from AD patients. We found there were differing degrees of decline in global efficiency, clustering coefficient, number of edges and transitivity in AD patients compared with healthy controls. The topology parameters of the evolution network tended toward those of the AD group. The results of SVM classification of the evolution network show that the evolution network had a greater probability to be classified as an AD patients group. A new biological marker for diagnosis of AD was provided through comparison of topology parameters between AD patients and healthy controls. The study of network evolution strategies enriched the method of brain network evolution. The use of SVM to classify the results of network evolution provides an objective criteria for determining evolution results.

## Introduction

Exploring brain development during the aging process and pathogenesis of the brain is an important part of human research [1], [2]. The human brain, as a control center, consumes a great deal of energy compared to its own weight [3]. However, the brain is fragile as occasional minimal trauma or variability of the nervous system can cause lifelong disease with little chance of recovery [4]. In modern societies, the incidence of stroke, traumatic brain injury and diseases resulting from brain injury are very common [5]. At present, there are also high rates of occurrence of mental disorders such as schizophrenia and Alzheimer’s disease (AD) [6]. Since these diseases seriously affect people’s lives, it is important to practically explore the internal mechanism and the structural and functional changes of neurological disorders of the brain [7].

Recent advances in theory and technology have been used to diagnose or treat brain disorders and further ongoing research. Current techniques for diagnosing brain diseases include electroencephalography (EEG) [8], magnetoencephalography (MEG) [9] magnetic resonance imaging (MRI) [10] and positron emission tomography (PET) [11]. Different research methods accompanied by diagnostic techniques have also emerged, for example, using dynamic causal modeling (DCM) and fMRI datasets, Grefkes et al. established a model of stroke patients and healthy controls to explore changes in motor areas of stroke patients [12]. Boord et al. analyzed changes in spinal cord injury (SCI) by independent component analysis (ICA) and EEG sources [13]. Other common research methods include SEM [14] and FNC [15].

However, as the brain itself is an extremely complex system [16], [17], it is very difficult to reveal the mechanism of brain incidence by studying unrelated parts of brain regions. Recently, complex networks have allowed for the exploration of brain development [18] and pathogenic mechanisms of brain diseases [19]. The topological parameters of the complex network can measure and assess the state of the brain network (including global efficiency, clustering coefficient, shortest path length, node degrees, small-world property, betweenness, transitivity and synchronization etc.) [20]. Using this method, many new reports have emerged in recent years. Dosenbach et al. predicted the brain development [18], Meunier and Chen explored the aging process of the brain [21], [22]. In addition, complex network theory has been used to research various nervous system diseases [23], [24]. In short, complex networks have penetrated all sub-disciplines of brain research.

Until now, the use of complex network theory to explore the development and changes in nervous system diseases has generally been applied to comparing one or several brain network topology parameters [19], [22], [23], [25]. Although this method can be used to find the quantization parameters of brain maturation or brain lesions, it is not suited for studying the mechanism of brain development or brain lesions. Network evolution is a method for generating a new network or for modifying existing networks by employing evolution strategies and control factors [26]. A study of evolution strategies and control factors can further an understanding of mechanisms underlying network formation. Evolutionary processes from network A to network B under various evolutionary strategies and control factors allow us to evaluate essential points of difference between network A and network B. This research method has obvious implications for studying brain development and the progression of brain disease.

Computational experiments and computer simulation technology can approach a rational model of the brain under laboratory conditions [27]. In the course of computational experiments, the system can be designed, analyzed, controlled, and integrated. The purpose of a computational experiment aims to realistically model actual systems set as the unique reference and standard to test whether simulation results are reasonable. Computational experiments can describe the multiple possible outcomes of evolution, resulting in a series of models which approximate more closely to the actual evolution of the brain. It will show that the use of computational experiments for brain network evolution is a viable research method.

As part of the study of brain network formation mechanisms, Vértes et al. have established a brain model using computational experiment and simulation technology similar to a rational brain network using the node degree and node distance as control factors. However, this model merely establishes a connection between the isolated nodes, while differences between actual brain development and disease processes remain [28]. Alzheimer’s disease (AD) is a disease caused by a variety of nervous system lesions, is a primary degenerative disease of the central nervous system and is characterized by cognitive deficits and prominent memory impairment [29], [30]. Recently, there have been great difficulties for early diagnosis and treatment of AD in patients [2]. In this study, the topology parameters of functional brain networks of AD patients and healthy controls were calculated and the differences between them analyzed. Then, reasonable network evolution strategies were formulated, and the evolution network hypothesized to approach that of the AD patient group was generated from a network of healthy controls using the computational experiment method. In order to assess the evolution results, the global efficiency, clustering coefficient, number of edges and the network transitivity of AD patients and healthy controls undergoing evolution was calculated. Finally, a support vector machine (SVM) [31] was used to classify the topology parameters of the evolution network.

## Materials and Methods

### Ethics Statement

This study was approved by the internal Institutional Review Board of Tongji Hospital and written informed consent was obtained from all participants; in the case of patients with dementia, consent was obtained from family members.

### Subjects

In this study, each subject received routine MRI examination to exclude prior neurologic diseases and multi-echo T2- and T2*-weight imaging to measure transverse relaxation rate R2. In addition, each subject underwent extensive neurologic and neuropsychologic examinations in clinic and was evaluated according to the NINCDS-ADRDA criteria for probable AD [32]. 10 right-handed AD patients (4male; age range: 52–81; mean: 65.6±9.88) and 11 right-handed healthy volunteers (4male; age range: 55–82; mean: 63.8±7.61) were selected to take part in this study. The AD patients were not symptomatic of other mental illness or brain damage. The specific circumstances of the patients are shown in Table 1.

**Table 1:** The clinical data of patients with AD (** represents MMSE score unfinished).

Number	Gender	Age	MMSE
1	M	52	23
2	F	61	15
3	M	60	16
4	F	79	23
5	F	55	18
6	M	73	25
7	F	69	**
8	F	67	14
9	M	81	13
10	F	59	21

### MRI Acquisition

During MRI acquisition, a 3T GE Signaxs scanner (General Electric) was used to acquire datasets. Specific scanning parameters are as follows: TR/TE = 2000/30 ms, FA = 90°, FOV = 24×24 cm^2^, Phase FOV = 1, Slice thickness 5.0 mm without space, Matrix = 64×64, NEX = 1, slices number: 33, Scan time 8 min. In order to achieve three-dimensional reconstruction and spatial registration, 3D SPGR (spoiled grass gradient recalled) was performed for each subject with the following parameters: TR/TE/TI = 6.5/2.1/400 ms, FA = 15°, FOV = 25.6×25.6 cm^2^, Phase FOV = 1, Slice thickness 1.0 mm without space, Matrix = 256×256, NEX = 1, Scan time 4 min 8 sec.

### Data Preprocessing

SPM8 (www.fil.ion.ucl. ac.uk/spm ) was used on MRI datasets for preprocessing. First, slice timing was used to correct for time-domain. The datasets was realigned to remove movement artifacts in the fMRI time-series. For in-group comparison, all the datasets were normalized to MNI space. Image datasets were smoothed by a FWHM of 4*4*4. Finally, datasets were drifted and filtered with 0.01 Hz–0.0 6Hz.

### Construction of Brain Functional Network

Covariates were removed after preprocessing, and time series of 90 brain regions for each subject, defined by the Automated Anatomical Labeling (AAL) [33], were extracted. Pearson correlation was used to establish the relationship between any two time series, so that a 90 * 90 correlation coefficient matrix was obtained for each subject. In order to compare the differences between AD patients and healthy controls in a wide threshold range, we set the threshold from 0.50 to 0.60, which is increased by 0.01 each step, to transform the correlation coefficient matrix in a binary matrix. The binary matrix was used to establish the functional brain network of the AD patients and the healthy controls.

### Network Evolution Rules

In a previous study, Vértes et al. simulated the brain network used by the control factors of node degree and the distance between the nodes [28]. In a complex network, node degree is the number of connections between the node and other nodes. It measures the degree of importance for the node and the number of connections in the network. Node betweenness is defined as the number of nodes that participate in the shortest paths of the network. Previous studies have illustrated that the brain is an optimized structure selecting the shortest path during information processing to save time and energy [34], [35]. Therefore, we believe that the betweenness which reflects the shortest number of paths may be more suitable for network evolution.

**Figure 1:**
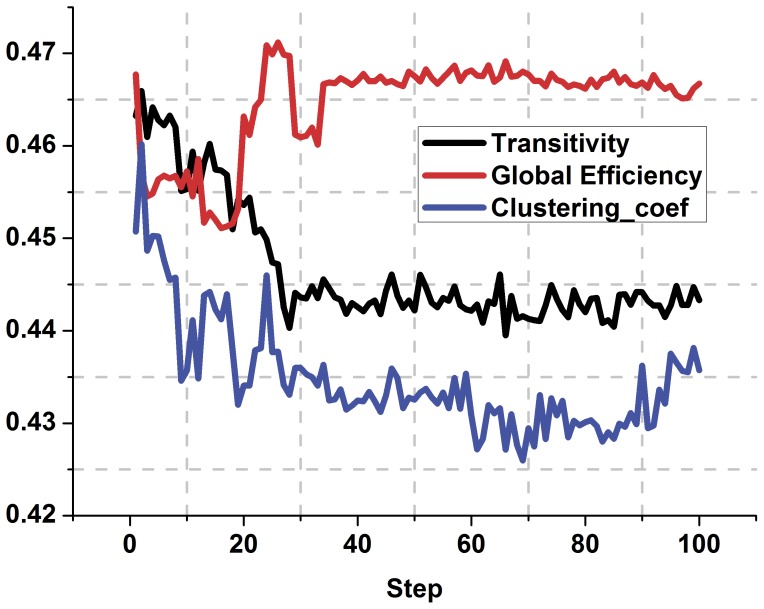
The trend of the network topology parameters during the process of evolution. The black line represents transitivity, the red line indicates the global efficiency, the blue line shows the clustering coefficient. Excluding the impact of random factors, the topology parameters can be considered stable for evolution steps up to 100.

For evolution processing from one network to another, connection weights (Equation 1) and disconnect weights (Equation 2) were formulated in this study:
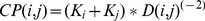
(1)

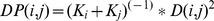
(2)Where CP(i,j) and DP(i,j) denote the connection weights and disconnect weights. Ki, Kj represents the i-th and j-th node betweenness respectively, D(i, j) represents the anatomical distance between the node i and j. In order to coincide with the individual differences and uncertainties in the disease process, a random factor R(i, j) is added to the evolution process. R(i,j) is a 90 * 90 matrix of uniform distribution between 0 to 1. During the evolution process, there is no connection between the nodes i, j, and a new connection is established between the i-th and j-th node only if CP(i,j)≥0.5 and R(i,j)≤0.03. If a connection exists between the node i, j, and simultaneously satisfies DP(i, j)≥0.5 and R(i, j)≤0.03, then i, j are disconnected. Otherwise, the state between i and j remains. Our results show that the topology parameters of evolution network remain stable when the evolution does not exceed 100 steps (Figure 1). Therefore, the evolution process stops when the evolution reaches 100 steps.

### The Network Topological Parameters

Global efficiency and clustering coefficient: The global efficiency and clustering coefficient of functional brain network of each subject and evolution networks under different thresholds were calculated and averaged within a group.

Number of edges: The number of edges of each network of each subject and of evolution networks under different thresholds were counted and averaged within a group.

Transitivity: Transitivity is the ratio of ‘triangles to triplets’ in the network [36]. For each network, transitivity was computed.

### Support Vector Machine Classification

Support vector machines (SVM) were first proposed in 1995 by Corinna Cortes [31]. SVM is based on the VC dimension of statistical learning theory and structural risk minimization principle. SVM seek the best compromise between model complexity (i.e., the learning precision of the specific training samples) and learning ability (i.e., the ability to identify any sample with no error). SVM posses many unique advantages in solving small sample size, nonlinear and high dimensional pattern recognition problems.

During the experiment, the topology parameters of AD patients and healthy controls were set as two categories. They were used as training samples to train SVM classification. Then, the topology parameters of evolution network were classified by SVM. If the topology parameters of network evolution were assigned to an AD group, the classification was accurate. Otherwise, the classification was wrong.

## Results

### Differences between the Healthy Controls and the AD Patients

**Figure 2:**
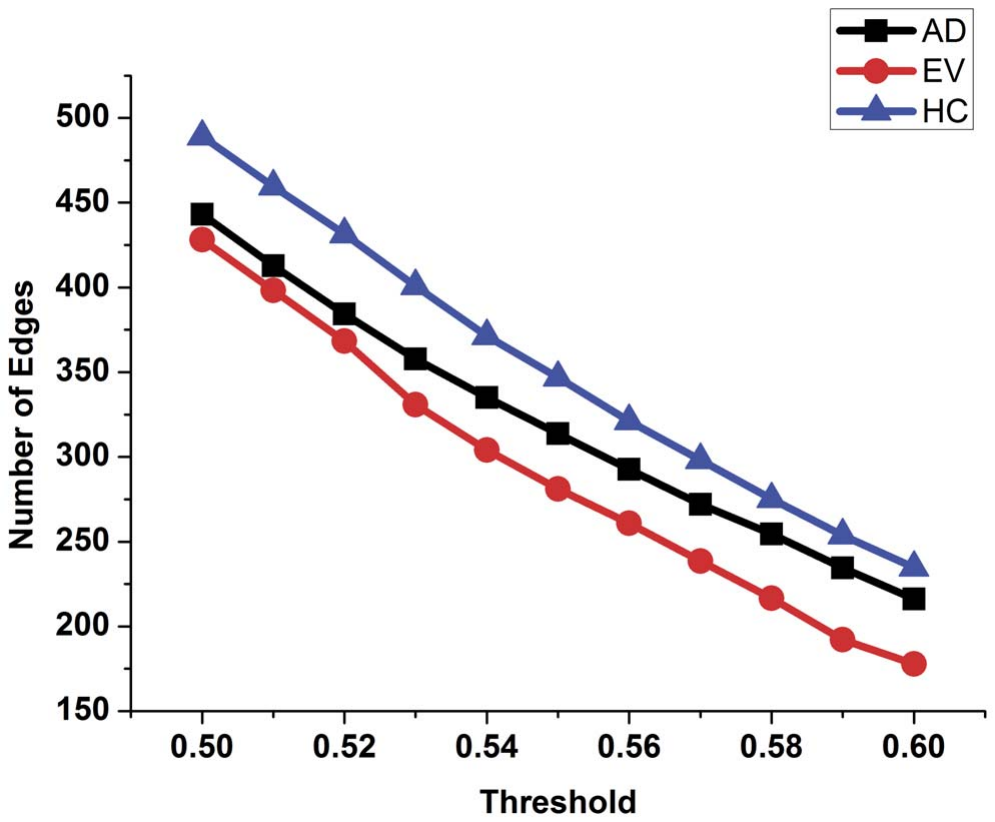
The number of edges of the functional brain network of healthy controls, AD patients and evolution under different thresholds. Blue triangle represents the healthy control group, black rectangle represents the group of patients with AD, the red dot represents the evolution network group.

**Figure 3:**
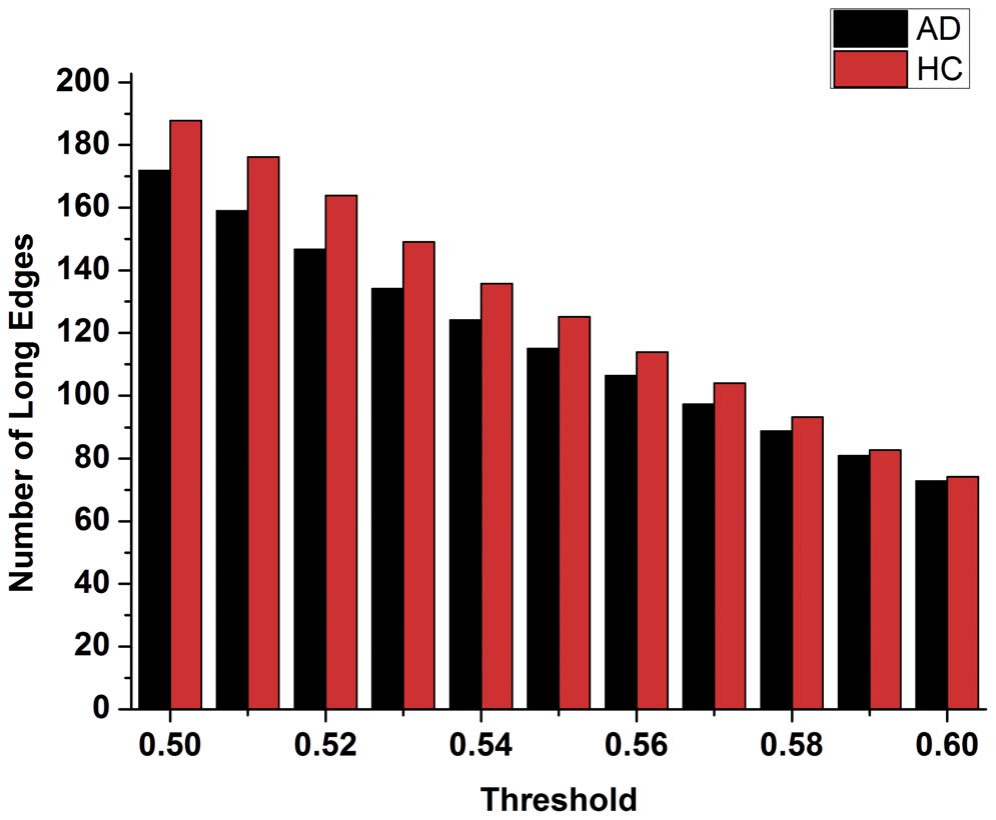
The number of long distance edges (>75 mm) of the functional brain network of the healthy controls and the AD patients. Red indicates the healthy control group, black indicates the patients of the AD group.

**Figure 4:**
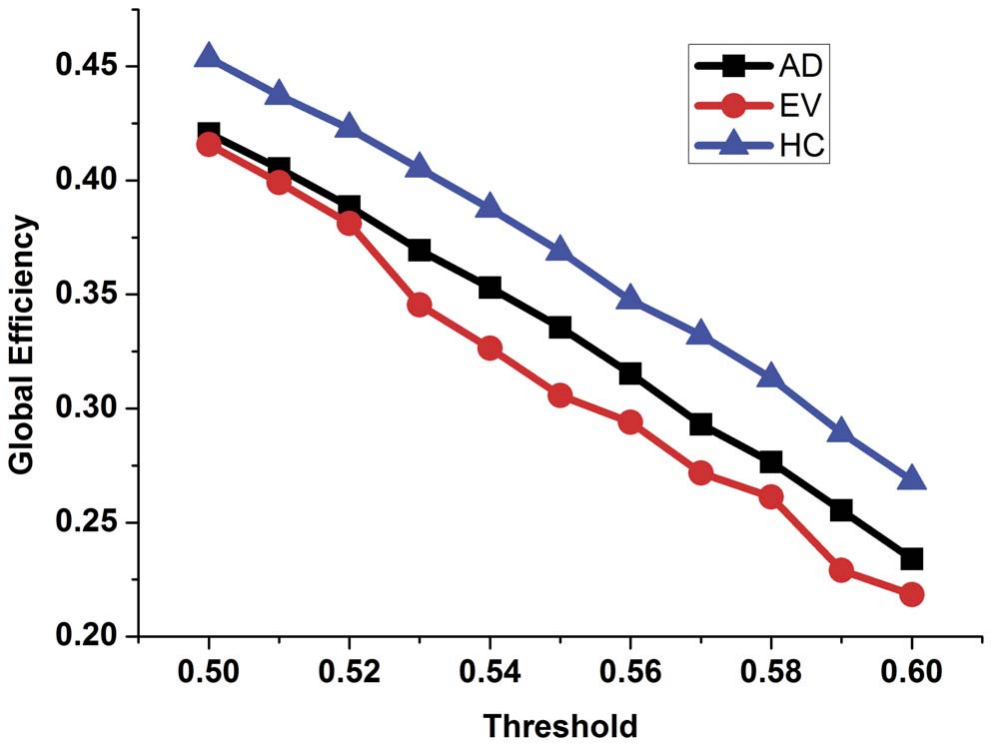
The global efficiency of the functional brain network of the healthy controls, AD patients and evolution under different thresholds. Blue triangles represent the healthy control group, black rectangles represent the group of patients with AD while the red dots represent the evolution network group.

**Figure 5:**
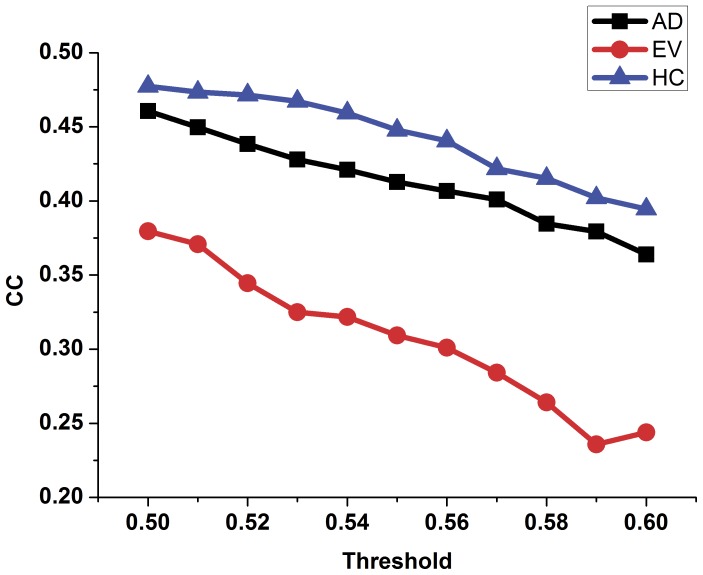
The clustering coefficient of the functional brain network of the healthy controls, AD patients and evolution under different thresholds. Blue triangle represents the healthy control group, black rectangle represents the group of patients with AD, the red dot represents the evolution network group.

**Figure 6:**
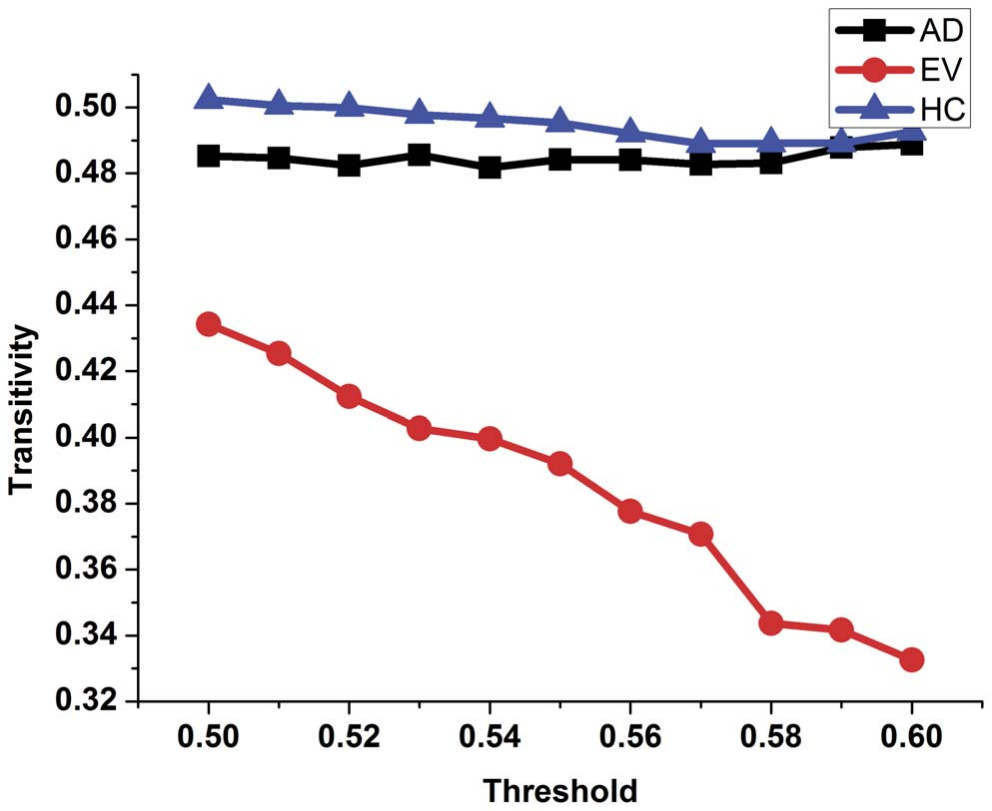
The transitivity of the functional brain network of the healthy controls, AD patients and evolution under different thresholds. Blue triangles represent the healthy control group, black rectangles represent the group of patients with AD, red dots represent the evolution network group.

In order to compare the topology parameters of the functional brain network between the AD patients and the healthy controls, the number of edges (Figure 2), the number of long-distance edges (Figure 3), global efficiency (Figure 4), clustering coefficient (Figure 5) and transitivity (Figure 6) of each functional brain network of each AD patient and healthy control were calculated.

In figure 2, the blue triangle represents the number of edges of functional brain network of the healthy control group under different thresholds, and the black square indicates the number of edges of the AD patients group. We found that the number of edges in the functional brain network of the AD group is less than that of the healthy group under different thresholds. Red represents the number of long distance edges of the healthy group, while black represents that of the AD group in figure 3. The number of long distance edges in the healthy group is more than that of the AD group. Blue triangles of Figures 4, 5, 6 represent the values of the global efficiency, the clustering coefficient and transitivity of healthy group under different thresholds. Black rectangles represent the values of the AD group. As can be seen from the figures, global efficiency, clustering coefficient and transitivity among the AD group were lower than among the healthy group.

### Comparison between the AD Group and the Evolution Group

The evolution networks were obtained from the binary network of healthy controls according to our evolution strategies under different thresholds. Topology parameters of the evolution network group are shown in Figure 2, 4, 5, 6 (red dot). From figure 2, we can see that the number of edges in the evolution network is closer to that of the AD group compared to the healthy group. The results of Figure 4 show that the global efficiency of the evolution network is closer to that of the AD group. Figure 5, and figure 6 imply that the clustering coefficient and transitivity of the evolution network are also closer to those of the AD group under wide threshold range.

### SVM Classification Results

The global efficiency, clustering coefficient, total number of edges, transitivity were set as eigenvalue for SVM classification. The accuracy of the classification is shown in Table 2. From table 2, the accuracy is above 55% of the total eigenvalue under different thresholds, and the average results of each characteristic are higher than 63%. The average accuracy of the eigenvalue for clustering coefficient and transitivity is 93.45%.

**Table 2:** Classification accuracy.

	globalefficiency (%)	clusteringcoefficient (%)	the total numberof edges (%)	transitivity (%)
0.50	55	82	64	91
0.51	64	82	64	91
0.52	64	82	64	91
0.53	64	91	64	91
0.54	64	100	64	91
0.55	64	91	64	100
0.56	64	100	64	91
0.57	73	100	64	91
0.58	64	100	64	100
0.59	64	100	73	91
0.60	55	100	73	100
Average	63.18	93.45	65.64	93.45

## Discussion

In this study, using the resting state fMRI datasets of the AD patients and the healthy controls, functional brain networks of each subject were established. The topology parameters within groups were calculated by averaging the dataset. The results illustrate that the density of the functional brain network of AD group is lower (Figure 2), and that the number of long distance edges of the AD group is less (Figure 3) than those of the healthy group under wide threshold range. The global efficiency, clustering coefficient, transitivity have different degrees of decline in the functional brain network of AD group. Using computational experiments and computer simulations, we set the node betweenness and distance between nodes as the evolution control factors and obtained a network evolution group from the functional brain network of the healthy group. The results suggest that the topology parameters of the evolution network are closer to those of the AD group than the healthy group. Such results imply that we can use network evolution to study the changes of functional brain networks in Alzheimer’s patients. Finally, SVM algorithm was used to classify the evolution results. The classification result shows that there was higher probability for classifying the topology parameters of the evolution network to the AD group. This proves the feasibility of the evolution method for the study of brain lesions.

A large number of studies have researched, using complex networks, the changes in functional or structural brain networks of patients with AD [19], [24], [37]. The results of Zhao et al. suggest that the global efficiency of the functional brain networks of AD patients declined compared to that of healthy controls [38]. Our results show that the global efficiency of AD patients was reduced under wide threshold range. Global efficiency reflects the ease of information exchange among network nodes [39]. A reduction of global efficiency in AD patients may mean that the efficiency of information exchange between the nodes in AD patients has declined [40]. Our results are consistent with previous studies.

The number of connection edges in a network is an indicator of the proximity of node connections [41]. In our study, comparing healthy controls, the number of edges in the functional brain networks of AD patients declined under different thresholds (Figure 2). This indicates that the connection density of the functional brain network of AD patients is lower than that of healthy controls. This means that compared to the healthy control group, the functional brain networks of AD patients is coupled less closely. Such a result may be a direct attribute of the efficiency decline of a network.

The average clustering coefficient is an indicator measuring the degree of coupling between small groups [20]. The decline in average clustering coefficient of patients with AD may imply degraded robustness and network optimization of functional brain networks in AD patients [42]. These results are consistent with previous studies of changes in functional brain networks of AD patients [43], [44].

The global efficiency of the complex network topology parameters was used to measure the average ease of network traffic. The clustering coefficient describes the proportion of neighbor nodes as mutually neighbors, that is, the degree of perfection of clique structure. Density of the network connection is an index to measure tightness between network nodes [20]. In our study, the global efficiency, clustering coefficient and the density of the functional brain network of the patients with AD all declined, implying that the efficiency of transmission of information between nodes was decreased. The physiological reasons for this phenomenon may be related to the reduction of synaptic connections of AD patients. Consequently, this may be due to the reduction of brain volumes in AD patients. Transitivity is the ratio of ‘triangles to triplets’ in the network [36]. It is another representation of proximity as well as a measure of the ease of information exchange between network nodes. The results illustrate that the transitivity of AD patients declined under wide threshold ranges. This may be the main reason for the decline of cognitive and memory function in AD patients [45]. Previous studies showed less functional connectivity between nodes of default mode network in AD patients [46]. This study does not research changes in the local nodes, however, it can be inferred that the reduction in the value of the topology parameters of AD patients was due to a decrease in the number of local connections.

In previous studies, the controlling factors of brain network evolution were node degree and node distance [28]. Node degree is a measure of the number of nodes directly connected to other nodes [41]. Node betweenness is a measure of the importance of the network information, material, or energy spent in the transmission process. Nodes with high node degree do not necessarily imply large betweenness. During our evolution, the node betweenness was set as one control factor. Previous studies showed that in order to save energy and time in the process of information exchange, transfer of information generally occurs through the shortest path among the nodes [47]. Therefore, from actual physiology, we have reason to believe that betweenness as a control factor is a reasonable criteria for the evolution of a brain network.

The distance between nodes is another index to measure time and energy required for information exchange between nodes [48]. The distance between nodes was set as another control factor during the evolution. We assume that the connection weights of the two nodes were inversely proportional to the distance squared, while the disconnection weights were proportional to the square of the distance between two nodes. To ensure that the value of disconnection weight increased during the process of the evolution, the control factor of the betweenness was taken as the sum of the betweenness of the two nodes, while the control factor of the distance was taken as the square of the distance between two nodes. This is in line with the general rules of the changes of a brain network with lesions. Our actual results verified this conclusion (Figure 2). Random disturbance factors were added in the evolution. We hypothesized that these small perturbations in the network evolution are more consistent with the actual process.

From the evolution results, we found that the number of edges in the evolution networks was closer to that of AD patients compared to the healthy controls. This proves that our evolution strategies can allow the functional brain network of the healthy controls to tend to that of the AD patients from the level of the density of the network. From the macro level, this shows that our evolution strategies and evolution method can control the evolution from the functional brain networks of healthy controls to that of patients with AD. In the evolution results, global efficiency, clustering coefficient and transitivity of evolution networks all tended to the parameters of the functional brain networks of the AD patient group under different thresholds. This result means that setting the distance between nodes and node betweenness as control factors in the process of evolution was a reasonable choice. Compared with the healthy control group, one reason for the decline of the topology parameters of the evolution network may be a decrease in connection density. Other reasons may include differing coupling distance between certain key nodes and other nodes.

A study using pattern recognition or classification standard to objectively assess the pros and cons of the evolution is not extant in the literature [28], [47], [48]. In this study, SVM was firstly used to classify the results of evolution. These results suggest that the topology properties of the evolution network have greater probability (average 93.45%) of classification than the AD patients group when clustering coefficient or transitivity are used as markers of classification. This suggests that our evolutionary strategies have a certain degree of reliability and rationality, and also show that we can obtain the functional brain network of the AD from that of healthy controls by using evolution rules.

As there is a certain impact on SVM classification accuracy due to the small size sample used in this paper, the conclusions of this study should be validated by a larger amount of data. At the present stage, we have not subjected the evolution network and the actual network of AD patients to an exact match in all details. However, our research has laid a foundation for future study.

## Conclusion

Using knowledge of complex networks and resting-state fMRI datasets, this study has researched the differences of the functional brain network between AD patients and healthy controls. The distance between nodes and node betweenness were first set as control factors in the evolution process. The results illustrate that the density of the network, the global efficiency, clustering coefficient and transitivity of AD patients declined compared to that of the healthy controls. Such results might identify new indicators for the early diagnosis of AD patients.

Evolution networks more similar to the functional brain networks of the AD patients were obtained from the functional brain network of the healthy controls. Our topology parameters of evolution networks were classified by SVM and our results show that the topology parameters of the evolution network can be classified into the AD patient group at a greater probability. This paper presents new evolutionary control factors for the study of brain network evolution, thus expanding the available methods for studying brain network evolution, and the use of SVM to classify the results of the evolution provides an objective standard for evaluating evolution results. We look forward to the use of our study as a basic platform for the evolution of different brain lesions in accordance with related research.

## References

[1] Wu K, Taki Y, Sato K, Kinomura S, Goto R, et al (2012) Age-related changes in topological organization of structural brain networks in healthy individuals. Human Brain Mapping 33: 552–568. 21391279 10.1002/hbm.21232 PMC6870030

[2] Biasutti M, Dufour N, Ferroud C, Dab W, Temime L (2012) Cost-effectiveness of magnetic resonance imaging with a new contrast agent for the early diagnosis of Alzheimer’s disease. PLoS ONE 7: e35559. 22532859 10.1371/journal.pone.0035559 PMC3332046

[3] Niven JE, Laughlin SB (2008) Energy limitation as a selective pressure on the evolution of sensory systems. J Exp Biol 211: 1792–1804. 18490395 10.1242/jeb.017574

[4] Lai SM, Studenski S, Duncan PW, Perera S (2002) Persisting consequences of stroke measured by the Stroke Impact Scale. Stroke 33: 1840–1844. 12105363 10.1161/01.str.0000019289.15440.f2

[5] Mintzopoulos D, Astrakas LG, Khanicheh A, Konstas AA, Singhal A, et al (2009) Connectivity alterations assessed by combining fMRI and MR-compatible hand robots in chronic stroke. NeuroImage 47: T90–T97. 19286464 10.1016/j.neuroimage.2009.03.007 PMC2720432

[6] Bauer CM, Jara H, Killiany R, Alzheimer’s Disease Neuroimaging Initiative (2010) Whole brain quantitative T2 MRI across multiple scanners with dual echo FSE: applications to AD, MCI, and normal aging. NeuroImage 52: 508–514. 20441797 10.1016/j.neuroimage.2010.04.255 PMC2907072

[7] Rowe JB (2010) Connectivity analysis is essential to understand neurological disorders. Front Syst Neurosci 4: Article 144. 10.3389/fnsys.2010.00144 PMC2953412 20948582

[8] Neuper C, Müller GR, Kübler A, Birbaumer N, Pfurtscheller G (2003) Clinical application of an EEG-based brain–computer interface: a case study in a patient with severe motor impairment. Clinical Neurophysiology 114: 399–409. 12705420 10.1016/s1388-2457(02)00387-5

[9] Bosboom JL, Stoffers D, Stam CJ, van Dijk BW, Verbunt J, et al (2006) Resting state oscillatory brain dynamics in Parkinson’s disease: an MEG study. Clin Neurophysiol 117: 2521–2531. 16997626 10.1016/j.clinph.2006.06.720

[10] Babiloni F, Cincotti F, Babiloni C, Carducci F, Mattia D, et al (2005) Estimation of the cortical functional connectivity with the multimodal integration of high-resolution EEG and fMRI data by directed transfer function. NeuroImage 24: 118–131. 15588603 10.1016/j.neuroimage.2004.09.036

[11] Nakayama N, Okumura A, Shinoda J, Nakashima T, Iwama T (2006) Relationship between regional cerebral metabolism and consciousness disturbance in traumatic diffuse brain injury without large focal lesions: an FDG-PET study with statistical parametric mapping analysis. J Neurol Neurosurg Psychiatry 77: 856–862. 16549415 10.1136/jnnp.2005.080523 PMC2117478

[12] Grefkes C, Nowak DA, Wang LE, Dafotakis M, Eickhoff SB, et al (2010) Modulating cortical connectivity in stroke patients by rTMS assessed with fMRI and dynamic causal modeling. NeuroImage 50: 233–242. 20005962 10.1016/j.neuroimage.2009.12.029 PMC8020334

[13] Boord P, Siddall PJ, Tran Y, Herbert D, Middleton J, et al (2008) Electroencephalographic slowing and reduced reactivity in neuropathic pain following spinal cord injury. Spinal Cord 46: 118–123. 17502876 10.1038/sj.sc.3102077

[14] Gates KM, Molenaar PC, Hillary FG, Ram N, Rovine MJ (2010) Automatic search for fMRI connectivity mapping: an alternative to Granger causality testing using formal equivalences among SEM path modeling, VAR, and unified SEM. NeuroImage 50: 1118–1125. 20060050 10.1016/j.neuroimage.2009.12.117

[15] Jafri MJ, Pearlson GD, Stevens M, Calhoun VD (2008) A method for functional network connectivity among spatially independent resting-state components in schizophrenia. NeuroImage 39: 1666–1681. 18082428 10.1016/j.neuroimage.2007.11.001 PMC3164840

[16] Passingham RE, Stephan KE, Kötter R (2002) The anatomical basis of functional localization in the cortex. Nature Rev Neurosci 3: 606–616. 12154362 10.1038/nrn893

[17] Sporns O, Tononi G, Kötter R (2005) The human connectome: A structural description of the human brain. PLoS Comput Biol 1: 245–251. 10.1371/journal.pcbi.0010042 PMC1239902 16201007

[18] Dosenbach NU, Nardos B, Cohen AL, Fair DA, Power JD, et al (2010) Prediction of individual brain maturity using fMRI. Science 329: 1358–1361. 20829489 10.1126/science.1194144 PMC3135376

[19] Supekar K, Menon V, Rubin D, Musen M, Greicius MD (2008) Network Analysis of Intrinsic Functional Brain Connectivity in Alzheimer’s Disease. PLoS Computational Biology 4: e1000100. 18584043 10.1371/journal.pcbi.1000100 PMC2435273

[20] Bullmore E, Sporns O (2009) Complex brain networks: graph theoretical analysis of structural and functional systems. Nat Rev Neurosci 10: 186–198. 19190637 10.1038/nrn2575

[21] Meunier D, Achard S, Morcom A, Bullmore E (2009) Age-related changes in modular organization of human brain functional networks. NeuroImage 44: 715–723. 19027073 10.1016/j.neuroimage.2008.09.062

[22] Chen ZJ, He Y, Rosa-Neto P, Gong G, Evans AC (2011) Age-related alterations in the modular organization of structural cortical network by using cortical thickness from MRI. NeuroImage 56: 235–245. 21238595 10.1016/j.neuroimage.2011.01.010

[23] Wang L, Yu C, Chen H, Qin W, He Y, et al (2010) Dynamic functional reorganization of the motor execution network after stroke. Brain 133: 1224–1238. 20354002 10.1093/brain/awq043

[24] Stam CJ, Jones BF, Nolte G, Breakspear M, Scheltens P (2006) Small-World Networks and Functional Connectivity in Alzheimer’s Disease. Cerebral Cortex 17: 92–99. 16452642 10.1093/cercor/bhj127

[25] Dickerson BC, Sperling RA (2009) Large-scale functional brain network abnormalities in Alzheimer’s disease: insights from functional neuroimaging. Behav Neurol 21: 63–75. 19847046 10.3233/BEN-2009-0227 PMC2872923

[26] Eisenberg E, Levanon EY (2003) Preferential Attachment in the Protein Network Evolution. Physical Review Letters 91: 138701. 14525344 10.1103/PhysRevLett.91.138701

[27] Kydland FE, Prescott EC (1996) The Computational Experiment: An Econometric Tool. Journal of Economic Perspectives 10: 69–85.

[28] Vértes PE, Alexander-Bloch AF, Gogtay N, Giedd JN, Rapoport JL, et al (2012) Simple models of human functional brain networks. Proc Natl Acad Sci USA 109: 5868–5873. 22467830 10.1073/pnas.1111738109 PMC3326510

[29] Qi ZG, Li K-CH (2008) Advance of diagnostic neuroimaging in Alzheimer’s disease. Int J Med Radio 731: 329–333.

[30] Verret L, Mann EO, Hang GB, Barth AM, Cobos I, et al (2012) Inhibitory Interneuron Deficit Links Altered Network Activity and Cognitive Dysfunction in Alzheimer Model. Cell 149: 708–721. 22541439 10.1016/j.cell.2012.02.046 PMC3375906

[31] Corinna Cortes, Vapnik V (1995) Support-Vector Networks. Machine Learning 20: 273–297.

[32] McKhann G, Drachman D, Folstein M, Katzman R, Price D, et al (1984) Clinical diagnosis of Alzheimer’s disease: report of the NINCDS-ADRDA Work Group under the auspices of Department of Health and Human Services Task Force on Alzheimer’s Disease. Neurology 34: 939–944. 6610841 10.1212/wnl.34.7.939

[33] Tzourio-Mazoyer N, Landeau B, Papathanassiou D, Crivello F, Etard O, et al (2002) Automated anatomical labeling of activations in SPM using a macroscopic anatomical parcellation of the MNI MRI single-subject brain. NeuroImage 15: 273–289. 11771995 10.1006/nimg.2001.0978

[34] Klyachko VA, Stevens CF (2003) Connectivity optimization and the positioning of cortical areas. Proc Natl Acad Sci USA 100: 7937–7941. 12796510 10.1073/pnas.0932745100 PMC164691

[35] Cherniak C, Mokhtarzada Z, Rodriguez-Esteban R, Changizi K (2004) Global optimization of cerebral cortex layout. Proc Natl Acad Sci USA 101: 1081–1086. 14722353 10.1073/pnas.0305212101 PMC327154

[36] Fagiolo G (2007) Clustering in complex directed networks. Physical Review E 76: 026107. 10.1103/PhysRevE.76.026107 17930104

[37] Rodriguez G, Arnaldi D, Picco A (2011) Brain Functional Network in Alzheimer’s Disease: Diagnostic Markers for Diagnosis and Monitoring. Int J Alzheimers Dis 2011: 1–10. 10.4061/2011/481903 PMC3100570 21629749

[38] Zhao X, Liu Y, Wang X, Liu B, Xi Q (2012) Disrupted Small-World Brain Networks in Moderate Alzheimer’s Disease: A Resting-State fMRI Study. PLoS ONE 7: e33540. 22457774 10.1371/journal.pone.0033540 PMC3311642

[39] Boccalettia S, Latorab V, Morenod Y, Chavez M, Hwang DU (2006) Complex networks: Structure and dynamics. Physics Reports 424: 175–308.

[40] Wolz R, Julkunen V, Koikkalainen J, Niskanen E, Zhang DP, et al (2011) Multi-Method Analysis of MRI Images in Early Diagnostics of Alzheimer’s Disease. PLoS ONE 6: e25446. 22022397 10.1371/journal.pone.0025446 PMC3192759

[41] Réka Albert, Albert-László Barabási (2002) Statistical mechanics of complex networks. Reviews of Modern Physics 74: 47–97.

[42] Sporns O, Chialvo DR, Kaiser M, Hilgetag CC (2004) Organization, development and function of complex brain networks. TRENDS in Cognitive Sciences 8: 418–425. 15350243 10.1016/j.tics.2004.07.008

[43] He Y, Chen Z, Gong G, Evans A (2009) Neuronal networks in alzheimer’s disease. Neuroscientist 15: 333–350. 19458383 10.1177/1073858409334423

[44] de Haan W, van der Flier WM, Wang H, Van Mieghem PF, Scheltens P, et al (2012) Disruption of Functional Brain Networks in Alzheimer’s Disease: What Can We Learn from Graph Spectral Analysis of Resting-State Magnetoencephalography? Brain Connect 2: 45–55. 22480296 10.1089/brain.2011.0043

[45] O’Dwyer L, Lamberton F, Bokde AL, Ewers M, Faluyi YO, et al (2011) Multiple indices of diffusion identifies white matter damage in mild cognitive impairment and Alzheimer’s disease. PLoS ONE 6: e21745. 21738785 10.1371/journal.pone.0021745 PMC3128090

[46] Han SD, Arfanakis K, Fleischman DA, Leurgans SE, Tuminello ER, et al (2012) Functional connectivity variations in mild cognitive impairment: associations with cognitive function. J Int Neuropsychol Soc 18: 39–48. 22005016 10.1017/S1355617711001299 PMC3368801

[47] Kaiser M, Hilgetag CC (2004) Modelling the development of cortical networks. Neurocomputing 58: 297–302.

[48] Alstott J, Breakspear M, Hagmann P, Cammoun L, Sporns O (2009) Modeling the impact of lesions in the human brain. PLoS Comput Biol 5: e1000408. 19521503 10.1371/journal.pcbi.1000408 PMC2688028

